# What is Mine? Behavioral and Anatomical Dissociations between Somatoparaphrenia and Anosognosia for Hemiplegia

**DOI:** 10.3233/BEN-2012-110226

**Published:** 2013

**Authors:** Paola Invernizzi, Martina Gandola, Daniele Romano, Laura Zapparoli, Gabriella Bottini, Eraldo Paulesu

**Affiliations:** ^1^Psychology DepartmentUniversity of Milano-BicoccaMilanItaly; ^2^Psychology DepartmentUniversity of PaviaPaviaItaly; ^3^Cognitive Neuropsychology LaboratoryNiguarda Ca' Granda HospitalMilanItaly; ^4^IRCCS GaleazziMilanItaly

**Keywords:** Somatoparaphrenia, anosognosia, awareness, ownership

## Abstract

We describe the clinical manifestations and the lesion patterns of five patients with somatoparaphrenia, the denial of ownership for a paralyzed limb, who showed the rare dissociation from anosognosia for hemiplegia. Similar cases have been only occasionally cited in the literature with scanty descriptions of their symptoms and no detailed anatomical assessment. All patients had extrapersonal and at least mild personal neglect. The lesions pattern was mainly subcortical, with a significant involvement of the right thalamus, the basal ganglia and the internal capsule. A formal comparison between the anatomical pattern previously associated with anosognosia in a study performed in 2005 by Berti and colleagues, and the lesion distribution of each patient clearly shows that our pure somatoparaphrenic patients had a sparing of most of the regions associated with anosognosia for hemiplegia. The behavioral dissociation between SP and anosognosia for hemiplegia, together with this new anatomical evidence, suggests that motor awareness is not sufficient to build up a sense of ownership and therefore these two cognitive abilities are at least in part functionally independent and qualitatively different.

## 1. Introduction

Somatoparaphrenia [1] is a delusional belief whereby a patient feels that a paralyzed limb, usually the upper left one, does not belong to his body; the symptom is typically associated with unilateral spatial neglect[1] and most frequently with anosognosia for hemiplegia [see for a review ref. 2]. A possible difference between disownership delusions and selective anosognosia for hemiplegia [AHP; 4] was already pointed out by Gerstmann in 1942 [1], who introduced the term somatoparaphrenia to identify those patients who denied the ownership of an affected body part. Soon after, Critchley stressed that it is not always obvious how to distinguish between these two symptoms, frequently proposed as different forms or degrees of severity of the same defect [1,5], SP being a more severe manifestation of a body representation disorder. Indeed, while many pa-

[1] Interestingly, SP is systematically associated with extra-personal neglect (the inability to explore/represent extra-personal space) rather than with personal neglect (defined here as the inability to reach out the paralyzed limb while keeping the eyes shut) [see Table 1, in 2]. While the dissociation with personal neglect is a well documented one, there is only one case with a focal right insular damage [3] in which SP may had been present without any sign of spatial neglect: the techniques used to assess neglect by Cereda and colleagues [3] however, were not specified leaving a vast margin of uncertainty on whether neglect was completely absent in that patient.

ISSN 0953-4180/13/$27.50 © 2013 – IOS Press and the authors. All rights reserved

Table 1
Cases of dissociation between SP and AHP reported in the recent literature. Neurological, neuropsychological and anatomical characteristics of non-anosognosic patients with somatoparaphrenic delusions reported in the literature quoted in the Pub Med database [see also 2]. – No deficit; + mild deficit; + + + severe deficit. n.r. Not reported in the original paper. "Disownership": simple feeling of non-belonging in which, contrary to "attribution", the patient does not recognize a limb as his/her own one, but does not explicitly identify the believed owner of the deluded body part

| Clinical features | | Author and year of publication | | | |
|---|---|---|---|---|---|
| | | Halligan et al., 1993 | Halligan et al.,1995 | Daprati et al., 2000 | Moro et al., 2004 |
| Neurological deficits | Motor | + + + | ++ | ++ | + + + |
| | Sense of touch | + + + | + + + | + + + | + + + |
| | Visual field | + + + | + + + | + | + |
| | Proprioception | + + + | ++ | + + + | + + + |
| Neuropsychological deficits | Extrapersonal neglect | + | + | + | + |
| | Anosognosia | – (fluctuant) | – | – | – |
| | Personal neglect | – | + | – | + |
| Features of delirium | Object of delirious | left arm and leg | left foot | left hand | left hand |
| | Whose hand? | n.r. | cow | son | another patient |
| | Type of delirious | disownership | attribution | attribution | attribution |
| Brain regions involved in the lesion | Frontal lobe | | | | X |
| | Temporal lobe | | X | X | X |
| | Parietal lobe | | X | X | X |
| | Thalamus | | | X | |
| | Basal Ganglia | X | | | |

tients with AHP do not have SP, only very few dissociations between SP and AHP have been reported in the literature [6–12]. A recent study investigating the clinical features and the anatomical correlates of AHP and "disturbed sensation of limb ownership" (DSO) pointed out that additional abnormal attitudes towards the affected limb (including SP) are almost invariably (92% of cases) associated with AHP [13]. The authors suggested the existence of a continuum of symptoms of altered body awareness, and that self-attribution of actions and the sense of limb ownership may be "the front and the reverse side of one coin", both associated, at the neural level, with the same structure: the right posterior insula [13].

Evidence for a double dissociation of these two deficits has come from a study based on the WADA test: it has been found that 68% out of 62 subjects showed both anosognosia for the left arm's weakness and asomatognosia,[2] while only the 11% were only anosognosic [14]. Interestingly, 10% of the patients presented with an isolated asomatognosia, as when asked about their left plegic limb they manifested feelings of non belonging for it and attributed their limb to someone else.

Vallar and Ronchi [2] have recently reviewed the neurological and neuropsychological features of 56 brain damaged somatoparaphrenic patients described in the literature. For 44 cases detailed information concerning motor deficit and awareness for it was available. Among these, only 7 patients, among which 4 recent cases, showed SP apparently dissociated from AHP [6–12]; all of them had a right sided hemispheric lesion, mainly in temporo-parietal regions or in the subcortical structures (see Table 1). All of them suffered from a dense hemiplegia or hemiparesis, from a gross deficit in somatosensory sensation and proprioception, and a mild or severe deficit of visual field. In all cases in which the testing was reported, there was visuo-spatial neglect and personal neglect. With few exceptions, AHP was tested by means of an informal interview. Although patients are described as to have been aware of their motor deficit, nevertheless, from the description, it looks as if the level of awareness was frequently fluctuant. As the authors of these reports were not specifically interested in the identification of double dissociations between these disorders, description of AHP and SP is not always clear or detailed [6, 7,12]. For example it is not completely clear whether the reported sensations of disownership could be classified as somatoparaphrenia (defined as an elaborated, bizarre, repeated and refractory to correction delusion of disownership for a paralyzed limb, see [15]), or the comparatively less profound form of body schema disorder asomatognosia, more likely to appear in isolation from other disorders of body awareness [14].

To summarize, while there are indications that SP and AHP may be dissociable, the available evidence is

[2]The term *asomatognosia* is used to define, in comparison with somatoparaphrenia, milder forms of body ownership disorders ranging from simple feelings of an arm missing or fading away to a feeling of non-belonging without elaborated delusional contents [13,15,16].

based on cases with incomplete descriptions either of the SP symptoms or of the level of AHP. However, a detailed analysis of such dissociations may give some insights on the cognitive processes underpinning the sense of ownership and motor awareness, two key components of self-awareness. Their relationship is a current matter of debate in cognitive sciences and philosophy. In addition, a detailed description of the anatomical correlates of dissociated cases may also prove useful particularly if such anatomical patterns were supportive of a double dissociation between SP and AHP.

To explore these issues, we retrospectively examined the records of a sample of 75 right brain damaged patients who had an extensive neuropsychological assessment for personal and extrapersonal neglect and associated symptoms. We found five cases showing disownership delusions without anosognosia for their left hemiplegia. These cases are the object of a detailed description here.

## 2. Methods

In order to search for cases of dissociation between SP and AHP, we retrospectively evaluated the clinical examinations and verbal reports of a series of patients who had been admitted at the Neurological Ward of the Niguarda Ca' Granda Hospital in Milano, and for whom a specialist neuropsychological consult had been asked.[3]

We selected a group of 5 patients with pure somatoparaphrenia dissociated from AHP. Selection criteria were: (i) evidence of a single, vascular lesion confined to the right hemisphere, as assessed by inspection of C.T. scans; (ii) no history or evidence of previous cerebrovascular disease, dementia or psychiatric disorders; (iii) presence of delusion of disownership for an affected body part; (iv) absence of anosognosia for hemiplegia as assessed by the Bisiach's scale (score $< 2$) and/or the Nimmo-Smith scale (score $< 5$ to each item).

All patients were formally assessed for neurological and neuropsychological deficits, after giving their informed consent to be tested as soon as their clinical conditions allowed a cognitive examination (1–5 days after the stroke) and before leaving the stroke unit (6–35 days).

[3]Some of the assessed patients also participated in other wider research programs.

### *2.1. Neurological evaluation*

The standardized examination for motor, sensory and visual field deficits has been administered according to the procedure proposed by Bisiach et al. [18]. Proprioception has been investigated with the examiner placing the patient's plegic arm in 5 different positions (arm pointing towards, arm pointing up/down, arm pointing to the right/left) and asking him to place his own right limb in the same position, with his eyes closed.

### *2.2. Neuropsychological evaluation*

The index of global cognitive functioning has been measured through the Mini Mental State Examination (MMSE) [19]. Since the presence of neglect may affect the patients' performance in some MMSE subtests (e.g. sentence reading, attention/concentration, and figure copy), hence lowering the overall score for reasons different from generalized mental deterioration, we also include patients who presented a marginally low MMSE score (score $\geqslant 22$), but were correctly oriented in space and time.

The presence of spatial extra-personal neglect has been investigated by means of the Albert Line-cancellation [20], the Letter-cancellation tasks [21,22] and the Line-bisection task. A pathological performance on at least one of these tests indicated the presence of neglect.

Personal neglect was assessed according with the procedure proposed by Bisiach and colleagues [18].

### *2.3. Anosognosia for hemiplegia*

Awareness for contralesional motor deficits has been explored by means of the four-points scale by Bisiach and colleagues [18]. The patient scored from 0 (in case of full awareness of the deficit) to 3 in case of severe anosognosia [18]. In some cases, patients have been also asked to evaluate their current ability of execution of a set of bimanual tasks by using the diagnostic test of unawareness of bilateral motor task abilities in anosognosia for hemiplegia as proposed by Nimmo-Smith and collaborators [23].[4] We reported the mean value of the patients' subjective evaluation of motor capabilities of performing the different proposed actions. According to the scale, a score of five or more has to be considered as an overestimation of one's own abilities

[4]Only for patients tested after 2005.

### 2.4. Somatoparaphrenia

Somatoparaphrenia was investigated by interviewing patients about the selective delusion confined to the contralesional limb including the following questions: "What is this? Whose hand is this? Where is your hand? Why is a foreign hand here?". Patients were considered as somatoparaphrenic in case they firmly denied that the arm belonged to them and/or attributed it to someone else providing elaborated, bizarre, persistent and refractory-to-correction explanations of their delusion [15]. Any other milder forms of body ownership disorders such as feelings of arm missing or fading away, were classified as asomatognosic symptoms as proposed by Feinberg and colleagues [15,16] but have not been considered in our study as our focus of interest was the association between AHP and clear-cut SP.

We considered as pure somatoparaphrenic patients, those subjects that presented with firm and refractory to corrections delusions of disownership of the affected body part, while being totally aware of their hemiplegia (Bisiach score $< 2$ and, if available, Nimmo-Smith score $< 5$ to each item).

### 2.5. Lesions mapping

For the selected patients with pure SP, cerebral lesions have been mapped using the MRIcro software application (www.mricro.com) on each corresponding slice of a standard M.R.I. template (a T1-weighted template M.R.I. scan from the Montreal Neurological Institute (www.bic.mni.mcgill.ca/cgi/icbm view) appropriately rotated to conform with the patient's C.T. scan acquisition angle (see the detailed mapping procedure in Gandola et al. [17]. The percentage of damaged voxels inside each brain regions of interest of the A.A.L. template of MRIcro has also been calculated.

Given the sample size of these series of pure SP patients, it was impossible to perform any statistical comparison with any control group of patients.

To provide an indication of the distribution of the brain lesions, we created a lesion plot overlay made by superimposition of the regions of interest (ROIs) of each patient lesion map and we isolated from it the set of brain regions that were constantly damaged in all of them pure SP patients.

The presence of SP in isolation from AHP would suggest at least a certain degree of anatomical independence of the two symptoms with the sparing in pure SP of certain brain structures previously associated with AHP.

To test this possibility, we assessed the degree of anatomical congruency of the brain lesions associated with cases of selective SP, with the results of a previous study from our group in which the anatomical patterns of AHP were investigated [24]. To this end, the ROI of the region commonly involved in all five patients and the single ROIs of each patient lesions were superimposed on the statistical map results of Berti and colleagues [24]: as the data were complying to the same stereotactic space, for each SP patient, it was possible to assess congruencies and dissociations in comparison with the AHP lesion pattern. The same was done with the set of brain regions that resulted to be constantly involved in all of the pure SP patients.

## 3. Results

Twenty four out of 75 patients with extrapersonal and/or peripersonal neglect presented with AHP for left hemiplegia, 7 of these AHP patients were also somatoparaphrenic. Interestingly, 5 patients showed SP dissociated from AHP[5] (see Table 2). These patients are the object of a detailed description here.

### 3.1. Case report 1

Patient CT was a 60 years old right-handed woman who worked as a school caretaker (educational level: 5 years). She was admitted to our Stroke Unit because of an ischemic stroke in the vascular territory of the middle cerebral artery, as assessed by C.T. scan. The first neurological examination, which was executed 5 days after the stroke, revealed extinction to double visual stimulation, left complete haemianestesia and a severe motor deficit of the left upper and lower limbs, with loss of proprioception. CT had also a severe extrapersonal neglect, neglect dyslexia and a mild personal neglect (see Table 2). The patient was fully aware of not being able to move her left arm and was able to detect motor errors. Furthermore, she was aware of not perceiving touches on the left hand. However, she continuously complained of a "foreign, strange and short hand" in her bed abandoned there by someone else.

Before she left the hospital (10 days after the onset), we could briefly re-assess her and registered an unvaried neurological examination with the same disownership sensations.

[5]Two of these patients (CT and GB) have been also included in a previous work focussed on the anatomical patterns underlying SP where no distinction was made between pure cases and cases with AHP [17].

Table 2
Features of the patients. Neurological, neuropsychological and anatomical characteristics of CT, GB, MA, AS and CP

| Demographic, neurological and neuropsychological features | Patients with somatoparaphrenia | | | | |
|---|---|---|---|---|---|
| | CT | GB | MA | AS | CP |
| Age | 60 | 69 | 71 | 84 | 70 |
| Sex | F | F | M | F | M |
| MMSE | 22,27 | 28 | 22 | 22 | 27 |
| Extrapersonal neglect | + | + | + | + | + |
| Personal neglect | 1 | 2 | 2 | 2 | 3 |
| Anosognosia for hemiplegia* | 1 | 1 | 0 | 0 | 0 |
| Anosognosia for hemiplegia § | 0 | 0,5 | 0,5 | 0,5 | 0 |
| Motor deficit | 3 | 3 | 3 | 3 | 3 |
| Deficit of the sense of touch | 1 | 3 | 3 | 1 | 3 |
| Visual field deficit | 3 | 3 | 1 | 3 | 3 |
| Proprioception deficit | + | + | + | + | + |
| Lesion site | right thalamus/BG | right frontal, temporal, insular, parietal regions | Right thalamus/BG | right temporo-occipital regions, thalamus / BG, internal capsule | right thalamus, BG and internal capsule |

*: Bisiach's scale [18]. §: Nimmo- Smith test (mean score) [23]; + = Presence of the symptom. 0-1-2-3 = Scores at the Bisiach's Scale; 0 = no deficit; 3 = severe deficit; for AHP: score 1 simply indicates that the patient is not aware of the occurrence of a stroke but knows about his/her paralysis. MMSE = Mini Mental State Examination; scores are reported corrected for age and level of education. BG = Basal Ganglia.

### 3.2. Case report 2

GB was a 69 years old right-handed woman who suffered a hemorrhagic subcortical stroke involving the right thalamus, hippocampus, basal ganglia (caudate, putamen, pallidum) and the deep white matter as documented by a C.T. scan. We assessed her 3 days after the stroke, and we detected a dense hemiplegia of the left arm and leg, hemianopia, a severe hemianestesia and a complete loss of proprioception. GB was correctly oriented in space and time and her MMSE score fully in the range of normality. Her neuropsychological evaluation was also adequate for linguistic functions and memory. She had extrapersonal neglect, moderate neglect dyslexia on a sentences reading task and a moderate personal neglect. GB was unaware of her hemianopia, while fully aware of her motor impairment: when asked to execute actions with the left arm, she suddenly detected failures with no hesitations. She was also completely aware of her somatosensory loss that triggered somatoparaphrenic delusions ("I can not feel touches, thus this can not be my hand"). GB often woke up in the night calling the nurses, scared for the presence of a foreign hand on her stomach, which she attributed to her niece Nadia, to a nurse or a patient previously admitted to the hospital in the same bed.

At the second evaluation, 8 days after the stroke, GB neurological deficits were not improved. Again, her awareness for motor impairment was complete, she always detected her failures when actual execution of actions was required and she was able to ascribe them to their real cause. The firm sense of non-belonging for the left hand with attributions to other people was unvaried.

### 3.3. Case report 3

MA was a 71 year old man (education level: 16 years) who suffered a hemorrhagic subcortical stroke involving the right thalamus, the hippocampus, the basal ganglia (putamen, pallidum and caudate nucleus) and the deep white matter, as assessed by a C.T. scan.

The neurological evaluation (5 days after the stroke) revealed a severe hemiplegia of the left arm and leg, hemianestesia, loss of proprioception and extinction to double visual stimulation. The MMSE score was slightly under the criterion for normality because of deficit in copying and reading due to extrapersonal neglect. MA also showed a moderate personal neglect.

The patient spontaneously complained and asked information about his left hemiplegia. On the contrary, he was anosognosic for his hemianestesia. Interestingly, when asked about the ownership of the left plegic hand, he often ascribed it to the examiner, while affirming that his "real" left hand was placed on his stomach.

At the follow-up (7 days after the stroke) no changes were detected in somatoparaphrenic symptoms, while eleven days after stroke the delusions had disappeared.

### 3.4. Case report 4

AS was an 84 years old woman (education level: 11 years) who suffered a stroke in the right brain hemi-

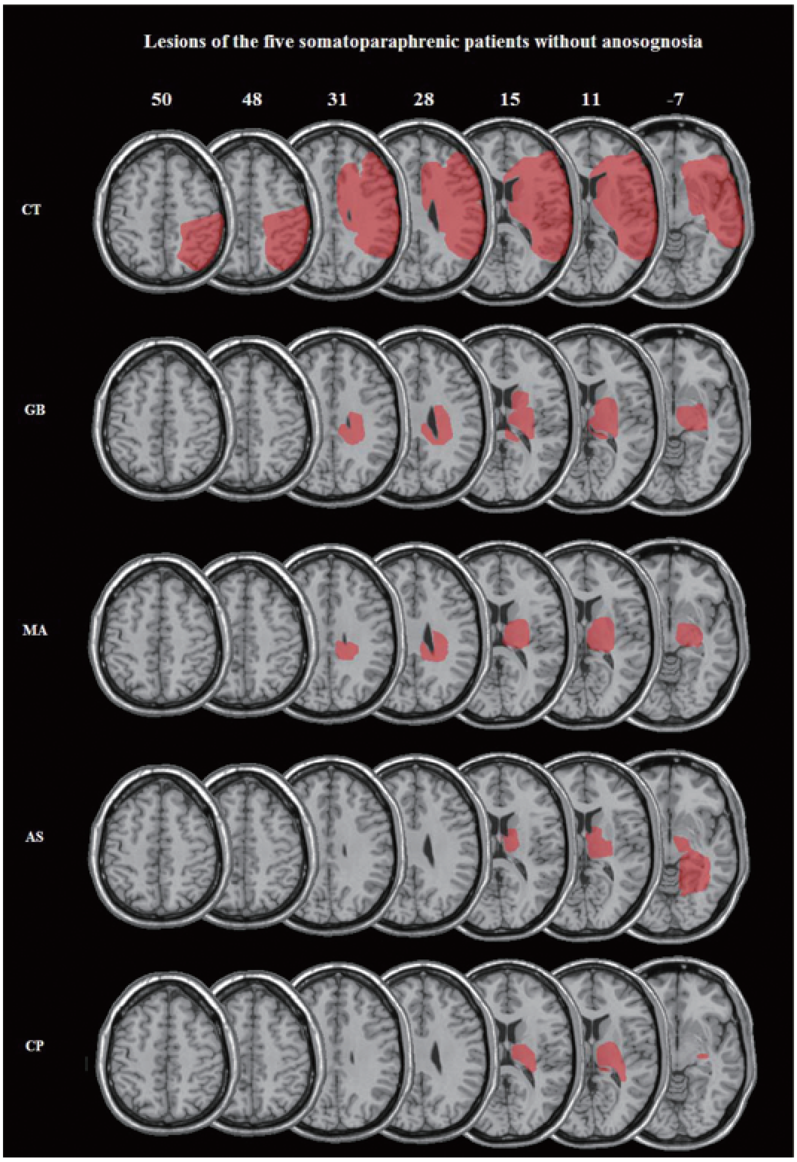

Fig. 1. C.T. scans of the five patients with somatoparaphrenia without anosognosia for hemiplegia.

sphere involving temporo-occipital regions, the thalamus, the basal ganglia and the white matter fibers of the internal capsule. We had the opportunity to test this patient 3 weeks after the stroke, but we had detailed clinical data available also for the acute phase. In the acute phase of the disease, AS had a severe hemiplegia of the left arm and leg, hemianestesia and haemianopia. A severe hemineglect was evident in all the performed tests. Her detailed case history described the presence of delusion about the ownership of the paretic limb, while there were no records of AHP. When we evaluated AS three weeks later, her total global cognitive functioning index (MMSE) was slightly below normality mainly because of the presence of a severe neglect, that invalidated tasks based on visuospatial exploration. AS also showed a severe personal neglect. At the neurological examination, only the tactile defect had improved and now she only showed extinction to double stimulation. She was fully aware of the left hemiplegia and of her visual field deficit. As her case history described disownership delusions in the acute phase, AS was interviewed about the sense of limb ownership. Again, these delusions emerged and the patients immediately denied the ownership of the left arm at the question

"whose hand is this?", affirming that the hand shown by the examiner was too fat, puffy and disobedient to her motor commands. Moreover, she asked the examiner to take away that "foreign" hand and replace it with her own "real" one, that someone surely had hidden away.

A further follow-up (5 weeks after the stroke) revealed no neurological ameliorations, AHP was still absent, while somatoparaphrenic delusion had been replaced by a milder form body ownership unawareness.

### 3.5. *Case report 5*

CP was a 70 years old man admitted to the stroke unit following a stroke mainly involving the right thalamic region and subcortical white matter. At the first neurological and neuropsychological assessment performed the day after the onset, CP was densely hemiplegic and also showed severe hemianestesia, haemianophia, and deficit of proprioception. He also had a severe personal neglect and extra-personal visuospatial neglect in all the proposed tasks. His MMSE was in the range of normality. When questioned about his problems, CP spontaneously complained about his hemiplegia, his inability of feeling touches in the left arm and of finding its position. As the examiner placed the paretic hand in front of CP's eyes, he suddenly attributed it to either a doctor or a friend. He was completely sure of this misattribution and when the examiner placed his own left hand near to the patient's one, he was totally unable to decide which one of them belonged to his body, maintaining that both of them belonged to the doctor. When presented with three hands (his own one and both the experimenter's hands), he persisted in attributing all of them to the examiner, in spite of the obvious nonsense of three arms in the same human body. During the following days, CP always remained fully aware of his neurological deficits; somatoparaphrenic delusions became fluctuant but persisted until six days after the stroke.

## 4. Anatomical results

The lesion plot overlay revealed the structures most commonly damaged in these patients (regions commonly damaged in 100% of patients): right thalamus, basal ganglia (putamen and globus pallidum), white matter of the posterior limb of the internal capsule (see Fig. 2). This pattern is consistent with that described in the study of Gandola et al. [17] and by Zeller et al. [25].

However, the same pattern is considerably different from the one reported for AHP [24] (see Fig. 2).

The comparison of each single lesion of our patients with the statistical map results of Berti and collaborators [24] revealed that 4 out 5 patients had no lesion overlap with regions associated to AHP in that work [24]. Only the lesion of patient CT overlapped, in part, with the cerebral structures previously associated with anosognosia: her lesion involved a large fronto-temporo-parietal cortical network and subcortical grey nuclei as well. However, her damage was clearly more ventral than the one described for AHP, with a remarkable sparing of dorsal motor and premotor regions, and dorso-lateral prefrontal cortex; the voxel count inside each of these regions showed the sparing of the 93% of voxels in the dorsal premotor cortex (BA 6), the 62% in BA 44, the 78% in the precentral gyrus (BA 4) including the hand-motor area, the 68% in the postcental gyrus, and the 93% of area BA 46. Interestingly, the right insula, a brain region that has been related to anosognosia by others [26], was extensively damaged (98% of voxels) and yet patient CT did not manifest signs of unawareness for hemiplegia (see Fig. 3) while having a clear-cut somatoparaphrenia.

## 5. Discussion

The sense of embodiment is a complex experience, which involves more than one distinct component in sensation and action: we are normally aware that "the body we inhabit is our own" [27] and that our limbs belong to us; this omnipresent sense is called sense of "body ownership". Awareness of the state of the motor system and the sense of being, rather than not, the cause of an action [28], are also a crucial aspect of our sense of "being us" [28].[6]

Even though these experiences have common elements, the underpinning neural systems, the way they operate and eventually interact are still far from being fully understood.

In normal circumstances, these experiences are strongly linked, even quite inseparable. Much to reinforce the intuitive notion that the underlying normal mental states should share cognitive causal mechanisms, the study of brain damaged patients has revealed

[6]In the domain of motor cognition, we limit our reflections on awareness for the state of the motor system without touching the domain of agency, the sense of awareness of who is the actor of an action; the present study has nothing to offer in this respect.

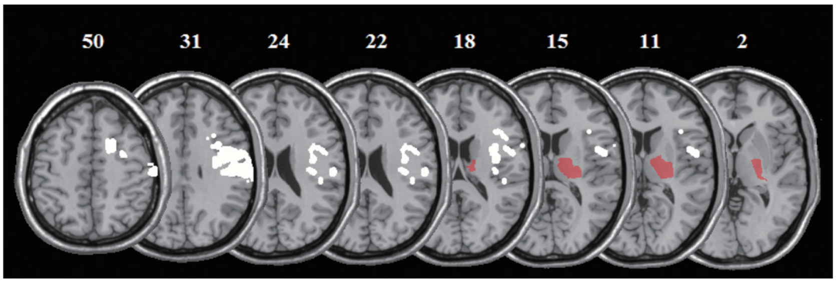

Fig. 2. The figures shows, in red, the center of overlap of the lesions of pure SP patients (the center is defined as regions commonly damaged in 100% of patients) and, in white, regions most commonly associated with AHP [24].

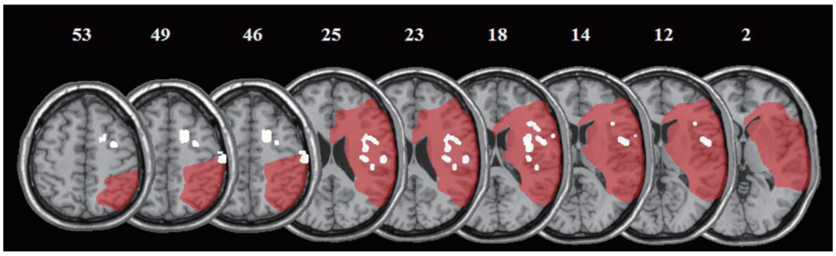

Fig. 3. In red, the lesion of CT, the patient showing also an extensive cortical damage, mapped in the stereotactic space and superimposed on brain regions (in white) involved in AHP [24]. In spite of the large cortical lesion, it is clear the sparing of some of the brain areas associated with AHP by Berti et al. [24].

a frequent co-occurrence of the disturbance of the sense of being able to move (e.g. AHP) and of the sense of body ownership (e.g. SP) (see [13]); this frequent co-occurrence of symptoms has justified the interpretation of AHP and SP as diverse manifestations of a common defect of body scheme representation, maybe due to insular damage [13,26].

The unitary interpretation of Bisiach accommodates, in functional terms, the multifarious repertoire of bodily based symptoms that one can observe in spatial neglect [29]. However, the unitary interpretation of certain syndromes does not necessarily imply that all symptoms should always coexist, nor it implies that they are explained by a single mechanism implemented in a single crucial brain region or network. Indeed, two disorders might be functionally independent impairments occurring together simply because brain damage tends not to be sufficiently specific (see [30]).

Recently, the sense of body ownership and the sense of being causally involved in actions have been studied in behavioral (e.g. [31–33]) and neurofunctional (e.g. [34,35]) experiments on healthy subjects. An "independence" model, which – contrary to the "additive" model – holds that the sense of agency and body ownership are qualitative different, has recently been supported by fMRI evidence showing different underpinning sets of brain regions, with no shared activations for these two component of the sense of self (see [27]). However, this study is based on the well known rubber hand illusion, an illusory perception of a prosthetic hand as part of the self: it remains to be established whether this experimental manipulation is sufficient to describe the human spontaneous sense of body ownership [25]. In addition, the sense of agency may not necessarily fully correspond to the ability to monitor the power of the motor system, the cognitive ability tackled by the interviews for AHP.

Our anatomical observations may provide an initial contribution to disentangle sensory and motor aspects of body-representation in the brain. An explicit comparison of the lesion pattern associated with AHP and SP in anatomo-clinical correlation studies with different groups of patients has not been attempted yet because of the rarity of pure SP patients. However, our preliminary anatomical evidence speaks in favor of dissociable neural networks for the manifestation of SP and AHP: a comparison of the anatomical pattern previously associated with AHP by Berti et al. [24] and the lesion distribution of each patient described here clearly shows that the pure SP patients had a sparing of most of the regions related to AHP.

Taken together, our behavioral and anatomical observations suggest that the frequent co-occurrence of AHP and SP might be most likely due to the limited specificity of naturally occurring brain lesions, rather than to a commonality of their central cognitive causal mechanisms.

Frontal premotor regions, together with the insula, seem to be crucial either for action representation [13, 24,26] and multisensory integration processes allowing the construction of a coherent body representation [25, 32,36]. However, while AHP seems to be tightly linked to this anterior damage affecting motor control processes embedded in the same neural network of the monitored primary function [24], anatomical data about SP show a more crucial involvement of grey subcortical structures and white matter bundles [16,17,25]. Indeed, the overlay lesion plot of our five pure SP patients clustered around subcortical grey nuclei and white matter fibers and does not share commonalities with the superimposed statistical map of regions associated to AHP [24], as confirmed by the comparison with each single patient's lesion.

A possible explanation of the frequent concomitance of AHP and SP is that neglect patients usually have extensive lesions that may affect frontal areas and subcortical grey and white matter causing both symptoms. Only in more rare cases, it may happen that, in the context of lesions leading to neglect and SP,[7] the premotor frontal cortex is preserved to such an extent to allow action monitoring and, thus, awareness of hemiplegia. The ideal candidates for this situation are patients with haemorragic lesions confined to subcortical regions with direct damage of the internal capsule and the thalamus, and a variable degree of deafferentation of the cortical mantle: a variable degree of dysfunction of the overlying cortex may explain the concomitant presence/absence of anosognosia.

### 5.1. *Explaining pure somatoparaphrenia?*

The putative anatomical mechanisms discussed above are insufficient to provide a comprehensive description of SP. Indeed, cases of SP associated with, or dissociated from, AHP propose challenging dilemmas on the functional mechanism whereby a patient feels a sense of non-belonging for the paralyzed limb while producing a delusional belief that a limb can correctly move (the canonical case of SP with AHP) or while correctly appreciating the paralysis of the limb, as in the dissociated cases. Let's concentrate here on the dissociated cases. According to Heilman's or Frith et al.'s models of motor control [38,39], the correct assessment of the motor status of a paralyzed limb should depend on the perception of a mismatch, by a comparator, between a motor plan and the lack of motoric consequences of the same plan. If this mechanism is valid for our dissociated cases, we should conclude that the correct assessment of the motoric status of a limb might not be sufficient to give a sense of ownership.

Further, it remains questionable which kind of feedback information is the patient using to appreciate the paralysis of the limb. Given the location of the lesion affecting the whole posterior limb of the internal capsule in all patients described here, a somatosensory feedback seems unlikely. On the other hand, are these patients making a better use of visual information and is their awareness limited to the "alien" limb through visual mechanisms? Would an interview of the patients while blindfolded modulate their reports? If this visual exploration hypothesis can explain the lack of AHP, why the same mechanism is insufficient to recognize the left limb as one's own?

Moreover, are patients talking about the same limb when assessing its motor power and the ownership?

Another interesting dissociation is the one between SP and personal neglect. In our series, only one patient had a severe form of personal neglect, being totally unable to localize his/her paralyzed limb. Four patients had a much milder form, being able to reach out the paralyzed limb after initial hesitations. Clearly, this represents a dissociation between some form of implicit knowledge, that permits the reaching behavior, and knowledge mediated by verbal descriptions, the one produced by the patients during the delusions of SP whereby *that* limb does belong to someone else.[8]

These and many others remain outstanding issues in this area, something that we were unable to assess for this time, given the retrospective nature of our study, a limitation that we overtly admit.

A detailed analysis of the patients' verbalizations and further manipulations of the patients' interview may help to shed some additional light on this particular dissociation as well.

For example, during the interview for the evaluation of SP we noticed that our patients seem to judge the motor capacities with reference to the foreign hand; patient CT said, "this hand, that does not move, is not

[7]For a more detailed discussion of mechanisms that may lead subcortical lesions to bring about "*cortical*" signs, see [17,37].

[8]It may be interesting to recall that personal neglect is normally tested with the eyes shut.

mine". An intriguing question is whether patients with SP and no AHP may refer to an alternative body schema of a foreign person, instead of their own corrupted bodily representation, to correctly evaluate actual motor performance. This possibility would be consistent with the evidence of Bottini et al. [40], in which the dense hemianestesia of a somatoparaphrenic woman recovered when she was instructed to report touches delivered to "her niece's hand", rather than to her own hand.

Further behavioral investigations, where one attempts to verbally modulate the patients' awareness of hemiplegia, may be helpful to explore this issue: for example, one could explore awareness for hemiplegia by asking to report about the "alien" limb as much as about "their" limb.

These and other experimental manipulations may help to further develop our understanding on SP and the functional mechanisms underlying the sense of body ownership.

## Appendix

*Patient CT verbalizations*

E: Close your eyes and tell me if you feel when I'm touching your hand. P: That's not my hand!!. E: What's the problem with it? P: It's not mine. I've already told it to the other doctor. Someone left it here. I don't know who he was. E: What did the doctor tell you? P: He asked me whose hand was this one, but *I don't know who attached it to my body*. E: Can you move this hand? P: *No, it does not move.* E: Isn't it a little bit weird to have a foreign hand with you? P: No! My hand is not like this! This is shorter, plus, *it does nothing*!! E: What is this hand doing here with you? P: Nothing. *It does not move. It does nothing*.

A second experimenter enters into the room and asks: Good morning C., can you repeat me your problem? Whose hand is this? P: I've already told it. *The problem is that this hand that cannot move is not mine*. It's not like mine. E: Raise your arms up, like me. Are you doing like me? P: No, I'm raising up the right arm only! E: Can you show me how you clap your hands? P: *I cannot, this hand does not move. But it is not mine*. You can take it away.

E = examiner; P = patient.

*Patient GB verbalizations*

E: What is this? P: it could be my hand. E: Could? Whose hand is this? P: Mine or yours. It's a female hand. *It's of the nurse*... but it wears my pajamas... it's strange. The examiner moves the pajamas away from GB sight and says: Whose hand is this?. GB touches the hand and says: I don't know. I can feel if you pinch the right one, but when I pinch that hand I can't feel I'm pinching. Obviously, *it's not mine. It's yours*. E: Where is your real hand? P: Here on the bed. E: If this hand isn't yours, can I take it away with me? P: Of course! If you want it, I will give it to you as my gift, since I have no need for it. *It doesn't work*. Maybe you'll be able to get it working. E: Are you sad about having that hand with you? P: Yes, a bit. I would like to understand why *it does not move nor work like the right one*. E: Do you want to move this hand away? Wouldn't you be sad without it? P: Yes, if it was mine, but it's not. E: Do you prefer that this hand was not so close to your body? P: yes, because *it's not mine*, *it doesn't look like mine. My hand is more thin and dry*. E: look at this hand, is it attached to your body or not? P: no. I don't know... I do not feel it. E: the nurses told us you woke up this night and called them, why? P: because there was this hand here and I thought that *Nadia forgot it* and I wanted to give it back her. She cannot work without it. Poor Nadia. E: Show me how you can clap your hands. P: *Impossible. Let's try... No, I'm not doing* it right, can't you see? I can make noise but *I'm not really clapping*. E: Look at me and do the same. Raise your hand up. P: It's the same. *I'm raising up only my right arm, the good one*.

E = examiner; P = patient.

*Patient MA verbalizations*

E: Why are you here? P: I had a stroke. E: How are you now? All right? P: No, *I can't move this arm.* E: Your left arm?. P: yes, but the same is for my left leg. *All the left side of my body is paralyzed.* Do you think I will ever be better? *I don't think I will be able to move them anymore*. E: Try to put your arms up in this way. P: *That hand does not move*. The examiner brought M.A. left hand in front of his face. E: What is this thing in front of you? P: A hand. E: Whose hand is this? P: *Yours!* E: Mine? Are you sure? P: Yes sure, whose hand is it supposed to be? E: and where is your left hand? P: On my stomach. Can't you see? E: So this hand isn't yours. P: No, my hand is on my stomach and cannot move. E: Whose hand could be this one? P: *Yours of course. Are you joking? Why should it be mine? My hand is different, not so heavy and it's not there, I always take it on my stomach.* E: this is my right arm and this is the left. It couldn't be mine. P: Then maybe it's the hand of the doctor. He surely needs it. Call him.

E = examiner; P = patient.

*Patient AS verbalizations*

E: how are you Miss S.? P: Not fine, *it does not work*. E: What does not work? P: This arm (she touches her left hand), *it does not move, it does not obey*. E: I understand, but if I touch you there, can you feel it? P: Yes, I do not know why but it does not obey. E: Why are you here? P: For a stroke. E: Try to get your hands in this way, as you were holding a tray up. She raises the right arm. E: Have you been able to do that? P: *No, the other hand* (*the left*) *is not working, it does not obey!!*. E: What's that? P: A hand of course. E: Whose hand is it? P: *I do not know*. E: Don't you know? Whose hand could it be? P: *Surely, it is not mine. Take it away*. E: If it is not yours, whose hand is it? P: *Someone working here examined me before and hid his hand into my bed as a joke! Give it back!* What a joke!! I would prefer my hand; this is too fat and puffy! E: Where is your real hand? P: I suppose *he took my hand away and gave me this bad one!* Go ask him!

E = examiner; P = patient.

*Patient CP verbalizations*

E: Hi, Mr. C. why are you here in this hospital? P: I had a stroke while I was on holiday. E: Which are your problems now? P: The main problem is with all the left part of my body. *I cannot feel it nor move it anymore*. E: Mr. C., look at this. What is this? P: *Your hand*. E: my hand? Are you sure? P: yes, of course. *It couldn't be mine*. E: Why? P: It looks groomer than mine. E: From zero to ten, how much are you sure that this is not your hand? P: Ten. E: How much are you sure that it is mine? P: Nine and a half. E: (after placing his left hand near the patient's one). Can you choose your own hand among these ones? P: *They are both of you*. E: (after placing both his right and left hands near CP's one). And now? P: They are yours. E: All of them three? P: yes. E: Don't you think they are too many hands for me? P: (smiling at the examiner). You are a polyp!

E = examiner; P = patient.
